# A blinded evaluation of privacy preserving record linkage with Bloom filters

**DOI:** 10.1186/s12874-022-01510-2

**Published:** 2022-01-16

**Authors:** Sean Randall, Helen Wichmann, Adrian Brown, James Boyd, Tom Eitelhuber, Alexandra Merchant, Anna Ferrante

**Affiliations:** 1 Centre for Data Linkage, School of Public Health, Curtin University, Kent St, Bentley, WA 6102 Australia; 2 WA Data Linkage Branch, WA Department of Health, 189 Royal St,, East Perth, WA 6004 Australia; 3 Department of Public Health, La Trobe University, Plenty Rd, Bundoora, VIC 3086 Australia

**Keywords:** Record linkage, Privacy preserving record linkage, Evaluation, Privacy

## Abstract

**Background:**

Privacy preserving record linkage (PPRL) methods using Bloom filters have shown promise for use in operational linkage settings. However real-world evaluations are required to confirm their suitability in practice.

**Methods:**

An extract of records from the Western Australian (WA) Hospital Morbidity Data Collection 2011–2015 and WA Death Registrations 2011–2015 were encoded to Bloom filters, and then linked using privacy-preserving methods. Results were compared to a traditional, un-encoded linkage of the same datasets using the same blocking criteria to enable direct investigation of the comparison step. The encoded linkage was carried out in a blinded setting, where there was no access to un-encoded data or a ‘truth set’.

**Results:**

The PPRL method using Bloom filters provided similar linkage quality to the traditional un-encoded linkage, with 99.3% of ‘groupings’ identical between privacy preserving and clear-text linkage.

**Conclusion:**

The Bloom filter method appears suitable for use in situations where clear-text identifiers cannot be provided for linkage.

**Supplementary Information:**

The online version contains supplementary material available at 10.1186/s12874-022-01510-2.

## Introduction

The task of privacy preserving record linkage (PPRL) involves identifying individuals from within and across datasets where these datasets have been encoded to ensure identifiers cannot be seen. Growing concerns about individual data privacy, along with an increasing demand for linked data from researchers has resulted in a burgeoning literature of new algorithmic approaches for providing PPRL [1]. Despite a plethora of documented PPRL algorithms, few have been operationalised into validated methods for use in real-world settings.

An emerging method is one using Bloom filters within a probabilistic matching framework. The Bloom filter data structure is used to hold encoded personal identifiers [2]. This method has the advantage of allowing tolerance for spelling mistakes and other small variations in identifiers.

An evaluation of this method by Randall et al [3]. showed that it was possible to achieve identical linkage quality to that found when carrying out linkage on un-encoded ‘clear-text’ data. While this evaluation highlighted the potential linkage quality that could be achieved with real world data, it was in some respects artificial, and still left a number of practical challenges unaddressed. These included the ability to validate incoming data correctly, set appropriate parameters for the linkage (vital for ensuring quality) and undertake appropriate quality assurance procedures post-linkage. A key remaining test for this method was to see not what results are technically possible to achieve in laboratory settings but to see what results could be expected in real word scenarios.

To address these challenges, we conducted a blinded evaluation where encoded administrative data was received and linked with no available knowledge of the ‘answers’, as in real-world scenarios.

## Methods

### Evaluation approach

This project represented a collaboration between two linkage units (organisations who each regularly undertake linkage for clients) - the Centre for Data Linkage (CDL) at Curtin University [4], and Western Australian Data Linkage Branch (WA-DLB), at the Department of Health, Western Australia [5].

**Fig. 1:**
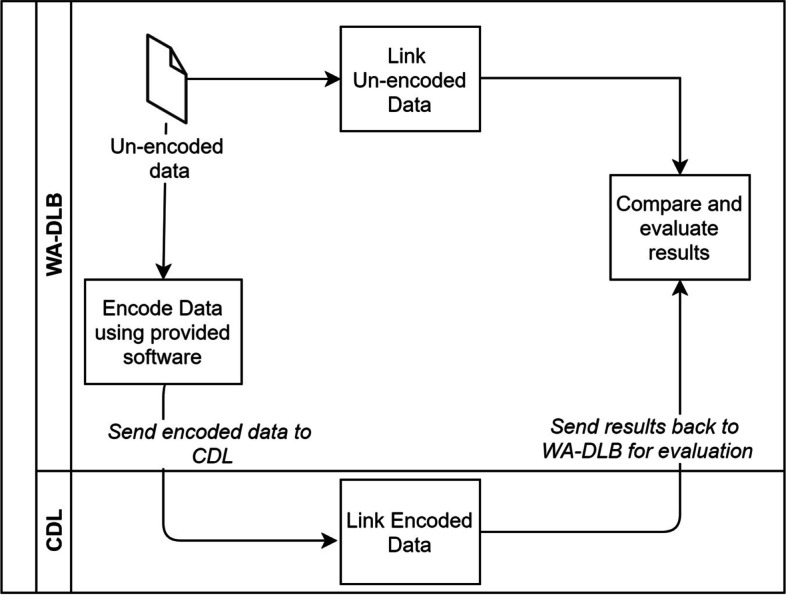
Data flows for PPRL evaluation

The overall approach and data flows are shown in Fig. 1. The un-encoded data used in the study were held by WA-DLB, where they formed part of their core linkage system. A portion of the data was extracted by WA-DLB and encoded into Bloom filters before being supplied to the CDL. The CDL then carried out the privacy preserving linkage. The results outlining which records belonged to the same/different individuals were then returned to WA-DLB. The WA-DLB separately conducted their own linkage on the un-encoded version of these same datasets, using custom linkage software [6]. Finally, the WA-DLB compared the two sets of results. With access to the un-encoded identifiers and the results in their core linkage system, they were able to identify whether particular record-pairs were correct or not, and to further investigate the reason for any discrepancy.

Ethical approval for this project was received from the Department of Health WA HREC (#2017/07). This approval included a waiver of consent.

### Datasets

Two datasets were used in the study. The first was an extract of all hospital separations 2011–2015 from the Western Australian Hospital Morbidity Data Collection (morbidity). The second was an extract of all Western Australian Death Registrations (mortality) 2011–2015. These datasets form part of the core data linkage system maintained by the WA-DLB, with updates to these collections regularly linked through their master system. There were 5,580,353 records in the morbidity extract and 68,955 records in the mortality extract. A summary description of the provided datasets is shown in Table 1.

**Table 1:** Percentage of missing values in each field in the two datasets

	Hospital Morbidity	Mortality
*Number of records*	*5,580,353*	*68,955*
**Fields (% missing)**		
Given Name 1	1.9%	0.1%
Given Name 2	50.6%	23.2%
Given Name 3	99.0%	93.5%
Surname	0.0%	0.0%
Sex	0.0%	0.0%
Date of birth	0.0%	0.1%
Address	0.0%	0.3%
Suburb	0.0%^a^	0.5%
Postcode	0.0%^b^	1.3%

^a^Increased to 0.1% after data cleaning

^b^Increased to 0.1% after data cleaning

### Privacy preserving linkage approach

The data was encoded for privacy preserving linkage using field-based Bloom filters; each identifier was encoded into a separate Bloom filter, with a standard probabilistic record linkage method used on these encoded identifiers. This approach has been described previously in the literature [3]; previous studies suggest it provides higher linkage quality than other privacy preserving methods [7].

The data was encoded at source by the WA-DLB, using the *LinXmart Simple Envelope Builder* [4]. This software is a simple desktop application that takes as input a dataset, a configuration file and a key, and returns as output an encoded (Bloom filter) version of the original dataset.

Names, address and suburb were encoded into Bloom filters, while all other fields were encoded with a single hash value. The Bloom filter encoding used bigrams with no padding, a Bloom filter length of 512, with 30 hashes per bigram for names, 20 for address and 25 for suburb. The number of hashes used was lower for suburb and address fields as the average length of these fields was longer. All hashes used HMAC SHA-2. Only the first 20 characters of the input fields were used in creating the encoded data. Some basic pre-processing steps occurred as part of the encoding process - these included removing whitespace, converting all values to lower case, and removing non-alphanumeric characters.

### Privacy preserving linkage methods

The encoded data was linked using the *LinXmart* software [4], using standard probabilistic methods [8].

Prior to linkage, the encoded data was validated. This involved confirming the number of records and fields received, checking the frequency of each field against expected values, and cross-checking to ensure the encoding across the two files was the same. The existence of the same encoded value across datasets for a particular field was taken as evidence of the same encoding being used. During validation, two address values were identified as being more frequently occurring than expected. These addresses were assumed to be placeholders such as ‘Unknown’ or ‘No Fixed Address’. These were removed. Two postcode values were almost solely associated with these addresses. These were assumed to be ‘dummy’ postcodes (e.g. ‘9999’) and also removed.

The data files were then linked – both within and between dataset matches were sought. All available fields (names, sex, dob and address) were used in the comparison process. Fields encoded using Bloom filters were compared using the Sørensen–Dice (Dice) coefficient. A default set of weights (m and u probabilities) were used; these had been developed and validated through project-based linkages previously carried out by the CDL. They are found in Additional file 1: Table 1.

To reduce the number of pair comparisons, only a subset of records were compared. This reduction was achieved by placing the records in each file into blocks so that only record pairs that agree on certain fields were compared. The same blocking strategy was used for the privacy preserving and clear-text linkages; this ensured that all differences in results reflected differences between encoded and non-encoded record comparison, rather than due to records which were simply not compared by one party. The blocking strategy was defined by WA-DLB based on what is used routinely in its core linkage activity. These blocks have been trialed and validated over many years to reduce false positive links, which are problematic in an enduring multi-set system [9]. The blocking strategy is found in Additional file 1: Table 2.

The acceptance threshold for matches was set by manual examination of a random sample of record pairs at particular scores; the threshold value of 20 was chosen. While no personal identifiers could be seen during this process, it was still possible to carry out some level of manual review/quality assurance by looking at the pattern of identifiers that matched between pairs of encoded records. It was left for the linker to use their judgement based on linkage experience to identify patterns of identifiers likely to be/not be a match.

After linkage, a number of quality assurance checks were carried out to investigate and potentially modify groups of records, such as those containing low-weighted pairs, those containing a large number of records, and those containing multiple death records. Record-pairs that appeared to contain male-female twins (all identifiers matching except first name and sex) were split, as were several groups with multiple death records. No other changes were made.

### Clear-text linkage methods

The un-encoded data was linked by WA-DLB using their in-house *DLS3* linkage software [6]. This software was specifically designed to merge incoming datasets into their ongoing repository of links. DLS3 is not typically used to link a small number of datasets to one another in isolation, rather, the software and the linkage strategies that are used with it assume the existence of a large body of existing links from a variety of data sources. For this evaluation, significant changes were made to how DLS3 handles and compares the data, so that a direct “one to one” linkage of the two datasets could be implemented.

The matching strategy used by the WA-DLB was designed to link these particular datasets, taking into account their specific character and idiosyncrasies. Separate matching routines were used for morbidity-to-morbidity comparisons, as compared to morbidity-mortality record comparisons. The matching process is relatively complex, with different sets of comparisons and processing rules for each individual block (see Additional file 1: Table 2 for the blocking strategy). These comparison rules focus on the individual fields not included in the block, and include approximate comparisons, comparison of parts of fields, and inversion comparisons (such as swapping of first and last names).

Following linkage, WA-DLB did not carry out any clerical review or quality assurance checks, and accepted all automated links produced by DLS3. Although this differs from WA-DLB’s typical linkage processes, these checks were left out to remove the “advantage” of undertaking a subjective plain text analysis outside of the DLS3 software.

### Method for evaluating quality

The evaluation was carried out on the linkage of morbidity to mortality data only; groups containing only morbidity records were not evaluated. Differences between the PPRL and clear-text linkage were examined. For each mortality record, the particular morbidity records that were linked to it in each linkage were compared to determine whether they were identical, or differences existed. Where differences existed, these were classified into those where clear-text linkage found additional morbidity records, and those where PPRL found additional morbidity records. These differences were assessed by manual inspection and comparison to their core linkage system to determine whether the additional link was correct or not, and the likely reason for the discrepancy.

## Results

There were 68,955 mortality records in this study; the morbidity records that linked to each of these mortality records in both the clear-text and PPRL linkages were compared, with key results shown in Table 2. Of the 68,955 mortality records linked in the study, for 99.3% (*n* = 68,478) the linkage results found with PPRL and with clear-text linkage were exactly the same. These 68,478 mortality records linked to 10,191 hospital morbidity records.

**Table 2:** Results of the comparison of clear-text and PPRL linkage

	n	%
Mortality records	68,955	100.0%
Links to morbidity records found by BOTH clear-text/PPRL	68,478	99.3%
Additional links found through PPRL only	48	0.1%
Correct	42	0.1%
Incorrect	6	0.0%
Additional links found by clear-text linkage only	432	0.6%
Correct^a^	383	0.6%
Incorrect^a^	55	0.1%

^a^For six mortality records, the clear-text linkage found both additional correct and incorrect links; these have been counted in both correct and incorrect categories

There were 479 (0.7%) remaining mortality records for which differences were found between the PPRL and clear-text linkage. For 48 of these, the PPRL method linked additional morbidity records not found in clear-text linkage, while for 432 the clear-text linkage found additional morbidity records not found through the PPRL method (for one mortality record, different morbidity records were linked by PPRL and clear-text methods and so it was counted in both the above categories).

All differences were manually examined by the WA-DLB, who had access to the un-encoded personal identifiers.

Of the 48 mortality records where additional morbidity records were found through PPRL but not clear-text linkage, the majority (88%, *n* = 42) were identified as being correct links. The reasons for the few incorrect PPRL links included the joining together of records belonging to twins, the joining together of a sibling pair, and the joining together of two unrelated individuals with similar names and dates of birth who resided at the same address (a hostel). An artefact of the incorrect joining of twins was the linking of multiple death records.

Of the 432 mortality records where additional morbidity records were found through clear-text linkage, the majority of these (89%, *n* = 383) were identified as being correct links. A large portion (67%, *n* = 291) of these additional morbidity records were missed by the PPRL method due to discrepancies in the blocking approach used by the two linkage units; although efforts were made to ensure these were identical, differences were identified after linkage upon review, which meant that the PPRL effort did not bring together all of the records that were expected, and thus certain records did not have the opportunity to link. This discrepancy was due to operator error and differences in implementation between the two systems, rather than an inherent characteristic of the PPRL method. Other causes of the links missed by the PPRL method included differences in recording and parsing of address details, and inconsistently recorded first names. For addresses, these included comparisons involving addresses with and without particular key words like UNIT, LOT and FLAT, and comparisons involving acronyms or other codes identifying the individual had no fixed address. An additional challenge was comparing address that contained a place name to those that did not (e.g. comparing ‘ACME AGED CARE HOME 1 JOHN ST’ and ‘1 JOHN ST’, or FULL ABORIGINAL COMMUNITY NAME and PARTIAL COMMUNITY NAME). For first names, the main challenge was identifying names with alternate spelling or diminutive forms (e.g. ELIZABETH and LIZ).

Of the 432 mortality records where additional morbidity records were found through clear-text linkage, 11% (*n* = 55) were identified as being incorrect links. These links were caused by incorrectly joining twins or cases of multiple death records incorrectly joining together, which were accepted due to WA-DLB’s intentional omission of standard post-linkage checks. There were six mortality records which contained additional morbidity records from clear-text linkage, where some were correct and some were incorrect; these have been counted in both the correct and incorrect tallies above.

## Discussion

The results achieved by the PPRL method were highly comparable to those returned from clear-text linkage. Identical results were found for 99.3% of groups created by either method; of the remaining 0.7% (*n* = 479), for approximately 20% (*n* = 97) the PPRL linkages were correct and for the remaining 80% (*n* = 425) the clear-text linkages were correct. Of these last 425, a sizable proportion were missed by the PPRL linkage due to user error (discrepancies in the blocking approach), rather than inherent issues with the PPRL method.

There were several elements of this evaluation that provide additional confidence in the results. Unlike previous evaluations of PPRL methods, here the truth set or ‘answer sheet’ was not available to those conducting privacy preserving linkage. All parameters were set based only on the available encoded data. In addition, there was no process of further ‘refinement’ of results based on feedback as to the actual answers. This differed significantly to the previous evaluation [3], which used parameters for the linkage calculated from the actual answers, and thus could not reflect a ‘real-world’ use case.

Differences remained between the two linkage approaches, aside from the use of encoded or clear-text data. The WA-DLB had expertise in linking these particular datasets, having developed a bespoke linkage strategy designed specifically to handle them, while the CDL used a generic strategy. The WA-DLB approach in this evaluation was entirely automated, while the CDL approach involved manual quality assurance checks to ensure the results were appropriate, which likely improved quality.

The investigation of linkage errors showed that there were some commonalities in the types of errors found through the privacy preserving and traditional linkage approaches. These included errors caused by incorrectly linking twins, errors caused by differences in address formatting, and differences in forms of first names. Twins represent a challenge for all methods of linkage, and this remains difficult to solve; indeed, the clear-text linkage also contained errors caused by twins because in-built checks using additional datasets outside the project scope which are used in the broader WADLS had been disabled. The errors caused by differences in address formatting and first names may be avoidable however, by pre-processing these fields (to convert address to a certain format, or remove specific keywords such as “LOT”, or convert diminutive names such as LIZ to the full ELIZABETH) prior to encoding into Bloom filters. This may be an avenue for further improvements in PPRL linkage quality.

While the evaluation here showed PPRL linkage quality to be essentially similar to that found with clear-text linkage, we do not expect this always to be the case. The nature of privacy preserving record linkage means it is more difficult to carry out quality assurance procedures and to identify instances where things have gone wrong. The resulting linkage quality may rely more heavily on the expertise of the linker than in clear-text linkage, where simple procedures such as manual review can easily identify processing errors. As such, the use of privacy preserving linkage techniques will always carry a greater risk of processing errors.

High linkage quality is valued to ensure the integrity of any results derived from the analysis of linked data. There is limited understanding of exactly how linkage error can affect research results or of the level of linkage quality needed to ensure the validity of results [10], although it is acknowledged that linkage error is heterogenous and particular populations can have reduced linkage quality [11]. Given the results found in this evaluation, it does not appear likely that privacy preserving record linkage would result in any degradation in research outcomes. However, further research investigating the relationship between linkage error and research results should explore this issue further.

In this evaluation, the encoded linkage strategy used the same blocking parameters as the clear-text linkage, which was provided by the clear-text linkers. This ensured all differences in results reflected differences in record-comparison between clear-text and encoded data. However, the relatively strict blocking criteria may have simplified the linkage for both parties, by reducing the available comparison space and therefore reducing the chance of false positives. It also had the disadvantage of not reflected real-world scenarios where blocking parameters are not provided.

This evaluation focused on the linkage quality aspect of PPRL methods, but another important consideration are the privacy implications. Bloom filter methods are not impervious to attacks, with applications to both field/key [12, 13] and record level Bloom filters [14, 15] (all fields combined in a single Bloom filter) documented in the literature. As a result, further modifications to Bloom filter encodings have been suggested [16, 17]. Work in this area is ongoing, although it should be noted that these modifications have an effect on linkage quality, with record-level Bloom filters unable to achieve as high linkage quality as found with field/key-level Bloom filters [7].

It is important that users understand the privacy aspects of the use of Bloom filters. In our model, Bloom filtered encodings are only to be released to a trusted third party, with significant legal and contractual safeguards and strong information governance in place. With these measures in place, Bloom filters are an important additional tool to reduce the risk of accidental of purposeful re-identification of individuals by the designated users who have access to them.

## Conclusion

These results demonstrate that PPRL methods can be used in real world applications. They can achieve very high linkage quality in real-world settings and should be considered as a solution, particularly where additional privacy protections are needed, or when data cannot be provided to linkage units in any other way. The nature of privacy preserving linkage means that there will always be a greater risk of processing error than with clear-text linkage and that care needs to be taken in the preparation and processing of encoded data.

## Supplementary Information

**Additional file 1: Table 1.** Match strategy used for PPRL. **Table 2.** Blocking strategy used for both PPRL and clear-text linkage.

## Data Availability

The data that support the findings of this study are available from WA Department of Health but restrictions apply as to the availability of these data, which are used under license for the current study, and so are not publicly available. Data are however available from the authors upon reasonable request and with permission of WA Department of Health.

## Abbreviations

CDL: Centre for Data Linkage
PPRL: Privacy preserving record linkage
WA: Western Australia
WA-DLB: Western Australian Data Linkage Branch
