# Supplementary material for: A blinded evaluation of privacy preserving record linkage with Bloom filters
Source: BMC Med Res Methodol. 2022 Jan 16;22:22. doi: 10.1186/s12874-022-01510-2 (PMC8761329; doi:10.1186/s12874-022-01510-2)
Supplement: Supplementary file 1 — Additional file 1: Table 1. Match strategy used for PPRL. Table 2. Blocking strategy used for both PPRL and clear-text linkage. [file 12874_2022_1510_MOESM1_ESM.docx]

**Appendix A**

***Table 1: Match strategy used for PPRL***

| **Field** | **Comparison Type** | **M-prob** | **U-prob** |
| --- | --- | --- | --- |
| Given Name 1 | Dice | 0.80 | 0.001 |
| Given Name 2 | Dice | 0.80 | 0.001 |
| Surname | Dice | 0.85 | 0.0002 |
| Year of birth | Exact | 0.97 | 0.012 |
| Month of birth | Exact | 0.85 | 0.08 |
| Day of birth | Exact | 0.85 | 0.03 |
| Sex | Exact | 0.9995 | 0.5 |
| Address | Dice | 0.5 | 0.0001 |
| Postcode | Exact | 0.8 | 0.01 |

***Table 2: Blocking strategy used for both PPRL and clear-text linkage***

| Block 1 | Surname + Given name 1 + Given name 2 + Date of birth |
| --- | --- |
| Block 2 | Surname + Given name 1 + Date of birth + Postcode + First 8 characters of address |
| Block 3 | NYSIIS value of surname + First 3 characters of given name 1 + First character of given name 2 + Date of birth |
| Block 4 | NYSIIS value of surname + First 3 characters of given name 1 + Date of birth + Postcode + First 8 characters of address |
| Block 5 | Surname + Date of birth + Postcode + First 8 characters of address |
| Block 6 | Given name 1 + Date of birth + Postcode + First 8 characters of address |
| Block 7 | Surname + Given name 1 + Sex + Day of birth + Month of birth + Postcode |
| Block 8 | NYSIIS value of surname + First 3 characters of given name 1 + Day of birth + Month of birth + First 6 characters of address |
